# Post-Pandemic Engagement of Youth in Virtual Environments: Reflections and Lessons Learned From the Development of a Youth Education Program

**DOI:** 10.1177/02724316231223527

**Published:** 2023-12-26

**Authors:** Jennifer Donnan, Rachel Howells, Dalainey H. Drakes, Lisa D. Bishop

**Affiliations:** 1 7512 Memorial University of Newfoundland, Canada; 2 Faculty of Social Sciences 6363 , University of Ottawa, Canada

**Keywords:** youth, youth engagement, research, COVID-19, virtual

## Abstract

The COVID-19 pandemic introduced new landscapes for research and public engagement participation. This shift was accompanied by significant challenges and unique opportunities for engaging youth as active participants and collaborators. This commentary will reflect on insights gained from conducting a variety of virtual youth engagement activities during the pandemic, within a rights-based and empirical approach. The team reflected on challenges, opportunities, and suggestions for engaging youth as participants and collaborators in research using virtual platforms. This commentary outlines opportunities for growth and challenges worthy of consideration for future virtual youth engagement activities. These considerations are put forth with the goal of upholding autonomy, diversity, and amplifying the voices of youth in research through virtual environments. Considering our insights on engaging youth, we hope to contribute to the expanding field of youth engagement, and advance future research that utilizes virtual modalities.

## Introduction

The importance of amplifying youth voices in research and program development contexts has come to light in recent years (Government of Canada, 2020). Empirical and rights-based frameworks have increased their focus on best practices to meaningfully engage youth (Lansdown, 2010; Lundy, 2007; Ozer et al., 2018), suggesting a shift in practice towards involving youth as active partners in the development and implementation of research and programs.

With the ever-changing research landscape, it is pertinent to consider methodological adaptations to achieve this goal within novel settings. While established youth engagement approaches (Bagnoli et al., 2010; Hawke et al., 2018; Powers et al., 2006) are still relevant, the COVID-19 pandemic accelerated the shift to virtual communication mediums, especially as over half a billion youth globally were forced to adapt to virtual learning (Cohen & Kupferschmidt, 2020). Further, average child and adolescent screen time increased by 52% relative to pre-pandemic reports (Madigan et al., 2022) with decreased face-to-face social interactions with peers for Canadian early adolescents (Gadermann et al., 2022). Consequently, youth are more familiar with social media and online communications platforms, highlighting a necessity to move youth engagement into these familiar and relevant spaces to optimize engagement. This commentary will reflect on our team’s experience virtually engaging with youth in early (age 13–14), middle (age 15–17), and late (age 18) adolescence during the COVID-19 pandemic as research participants and collaborators in the development of a youth substance use education program, targeted for youth aged 9 to 17 (Donnan et al., 2022; Drug Education, 2010). Youth were able to reflect on their current situation, as well as on their elementary years to describe what forms of knowledge, skills and education would have better prepared them for junior high.

**Figure 1.**
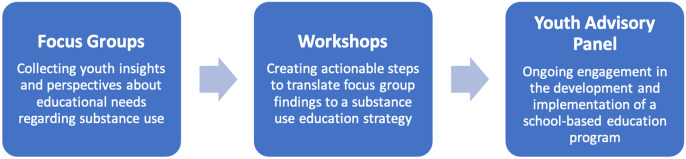
Youth engagement in the development of a youth education program.

Rights-based (Lansdown, 2010; Lundy, 2007) and empirical (Ozer et al., 2018) approaches are complimentary frameworks that portray youth as capable of creating knowledge. Moreover, these frameworks recognize that the inclusion of youth as active partners can impact the development of relevant programs and research. It is imperative for youth engagement that considerations be given to (i) equitable access to participation, (ii) representation of diverse populations, (iii) preservation of youth participant rights, (iv) environmental safety, (v) autonomy, and (vi) applications of outcomes to larger structural contexts (Lansdown, 2010; Lundy, 2007). Guided by these frameworks, our team engaged youth in the development of a youth substance use education program through a three-step process (Figure 1). We will reflect on opportunities for growth and challenges identified through this process that are worthy of consideration with a specific focus on recruitment, active engagement, and facilitation (Table 1).

**Table 1.** Opportunities, Challenges, and Suggestions for Engaging Youth as Participants in Online Research.

Opportunities	Challenges	Suggestions
Recruitment		
*Can attract a more representative sample:* 1. Online recruitment can reach urban and rural communities. 2. Broader reach to youths through social media and sharing of recruitment posts. 3. Increased awareness and credibility of the team’s ongoing research through social media. 4. More engagement in geographically diverse communities. 5. Less barriers to participation (e.g., scheduling, transportation). 6. Use of online platforms is free for participants.	*Recruitment can be hindered by:* 1. Social inequalities (e.g., Internet access, computer access, education, language). 2. Challenge to build relationships and trust with parents and guardians through online communication. 3. Challenge creating connections with youth organizations to share and promote recruitment. 4. Offering compensation can attract imposter participation; this is more challenging to screen in virtual environments.	1. Consider offering in-person or online information sessions to parents and organizations: *Anticipated outcomes:* - Increased awareness of research objectives - Increased knowledge of how the child may be involved, therefore more willing to provide consent. 2. Consider offering compensation in an ethical manner. *Anticipated outcomes:* - Increased opportunity for the involvement of youths from lower socio-economic backgrounds - Promotion of autonomy during research participation - Pre-plan ways to identify potential imposter participants through your recruitment strategy.
Active engagement		
*More creative and inclusive forms of participation:* 1. Multiple ways to communicate (e.g., chat, emojis, microphone). 2. More accessible for those with visual and hearing impairments. 3. Lessen power imbalances: Participants had power over where they could join, and had the choice to turn the camera on or off. 4. Familiarity and comfort with the online platform.	*Issues with rapport:* 1. When other participants do not turn their cameras on, it is harder for participants to openly converse. 2. Lack of face-to-face contact can hinder a warm environment. *Barriers to disclosure:* 1. Fear of family members or others overhearing conversations	1. Encourage participation through the chat function or use of emojis *Anticipated outcomes:* - Increased participation for those who do not feel comfortable speaking aloud in their home. 2. Overtly address the awkwardness of the zoom room when cameras and microphones are disabled, and how this hinders conversation. *Anticipated outcomes:* - Increased use of cameras and microphones - Better rapport between participants
Facilitation		
1. Including two young adult facilitators representing different genders (men, women, or gender diverse) to facilitate workshops, can help the youth, feel more comfortable. 2. Unique alternatives for sharing information (e.g., using the chat, responding with emojis, reactions, etc.). 3. Features like “raise a hand” ensured everyone was heard when they wanted to speak.	1. More difficulty identifying if participants are eligible for the study if they turn their cameras and microphones off. 2. Difficult to monitor the youth’s environments to ensure a safe space. 3. Some youth engage in multitasking during sessions. 4. Technology-related demands (e.g., poor Internet connection) may have interfered with sessions.	1. Communication prior to the start of the session to work through technological needs *Anticipated outcomes:* - Alleviates unexpected technological barriers during conversations. 2. Preface sessions with discussions about the importance of the youth’s voices in research *Anticipated outcomes:* - More present and intentional participation

### Recruitment

We recruited diverse perspectives with respect to gender, geographic location, age (early, middle, and late adolescence) and lived experiences, using a multi-faceted recruitment strategy. Paid ads through social media platforms were inexpensive and were directed to specific segments of the population. In addition to recruiting through popular youth platforms (e.g., *Instagram*), informative ads displaying our university affiliation on *Facebook and Twitter* helped build trust and parental support during the consent process. This also supported early adolescent recruitment by targeting parents of youth who were not yet using social media. However, we recognized that the sole use of online recruitment could hinder inclusivity due to inherent social inequities in access to the Internet (e.g., income, location), and the ability to use technology (e.g., education, language). These challenges were mitigated by engaging with established Community Youth Network leaders who were on the ground, engaging with youth in face-to-face settings. (The Community Youth Network is a program funded by the government of Newfoundland and Labrador to provide programs and services to youth right across the province). Using virtual communication platforms allowed us to reach geographically diverse youth and accommodate flexible scheduling, which would not have been possible if in-person attendance had been required. This helped promote equity and inclusion of diverse as well as historically underrepresented perspectives by providing a platform to amplify the voices of all youths.

To increase accessibility, we suggest investigating the availability of the Internet within target populations and offering spaces to engage virtually for those without access to the Internet. Additionally, it is important to engage with parental figures and community organizations, but this can be challenging in virtual spaces. Establishing contact with caregivers occasionally required additional coordination throughout our work, and we learned that a flexible approach to scheduling communication can maximize contact with caregivers who are initially hard to reach. To address caregiver apprehensions about providing consent, we propose offering information sessions to caregivers and organizations to increase awareness about research objectives and their child’s participation. A hybrid virtual and in-person recruitment strategy would help overcome some of these barriers and inequities. We encourage optimizing the use of the online environment by creating short video content posted to YouTube or TikTok that can be shared through email or social media. Inclusion of multiple mechanisms for addressing questions such as public chat functions or private emails and messages are important to timely respond to questions or address apprehensions. Despite efforts to mitigate caregiver apprehension, the requirement of parental consent may act as an unavoidable barrier to recruitment. Within our work, youth who were approaching late adolescence were often averse to this and halted contact after they were informed of such a requirement.

Monetary compensation ($25/hour) was provided to youth in accordance with guidelines for engaging with those with past or current lived experience (Taplin et al., 2019). Unfortunately, offering compensation also attracted some imposter participants. To mitigate this, we had youth register using a school-affiliated email address or provide another form of proof of identity. We also utilized online survey platforms (e.g., Qualtrics) for expressions of interest to allow for cross referencing IP address locations with their informed consent.

### Active Engagement

The use of virtual communication platforms allowed youth to safely engage as active participants as it facilitated support, cohesion, and relationality during a time of social distancing. This approach can also be valuable in non-pandemic periods as virtual communication is commonplace for today’s youth, promoting familiarity and comfort, while breaking down power imbalances between youth and facilitators that often accompany in-person engagement in research. For example, we promoted interactions through chat functions, raised hands, and the use of emojis. We recommend that facilitators prepare for this method of interaction in advance as it requires a different facilitator skill set.

Conversely, we acknowledge that virtual interactions may have hindered rapport and information sharing between participants. Youth had the option to keep their cameras off and were instructed to mute themselves until they needed to speak. Openly relating to the awkwardness of a virtual room when no one has their cameras on can help address speaking to a faceless room and encourages youth to turn on their cameras without applying pressure. Regarding content, late adolescents held more personal experience with the topics than early and mid-adolescents. To overcome this, we recommend encouraging youth to speak to observations of their peers if they lacked personal experience or did not want to share personal information. In our work, this promoted engagement regardless of age as we saw equal engagement efforts across the early, mid, and late adolescent age groups. Further, since discussion involved sensitive topics (i.e., substance use), it was important to acknowledge that participants may have apprehensive to share in fear of other people in their household overhearing them. To identify and help mediate any risk of harm, we suggest that facilitators, in a group of two, connect with participants before the start of sessions to review the information they may be asked to disclose and to inquire about the location in which they will participate. This will allow researchers to accommodate contextual and relational factors within the youths’ environments that may hinder their ability to speak candidly, and to suggest alternative settings if necessary.

The nature of our three-step engagement process was also critical in supporting youth as active research partners. Providing accessible opportunities to participate in research and later, to act as consultants and collaborators in the implementation of an education program is one pathway to meaningful engagement. Engagement that felt rewarding to the youth as they were able to see their insights translate to action. Throughout this process, we ensured that youth were viewed as experts on their own experience and were actively involved in key decision-making processes (Ozer et al., 2018). We fostered this environment and opportunities for youth by forming a youth advisory panel, providing a safe space for them to share their perspectives without fear of repercussion, and being a spokesperson for the adolescent voice when they were not present around the table.

### Facilitation

Including at least two facilitators for a session was also equally important for virtual focus groups as it is for in-person sessions to promote ease of discussion and for the protection of all involved (Bishop et al., 2022; Youth, 2010). While online platforms allowed for easy recording, a second person to manage and encourage chat conversations prompted active engagement.

Ethnoculturally diverse young adult facilitators worked together in gender-balanced pairs (e.g., woman-man) to lead groups to increase comfort and relatability for participants by using vernacular language unique to youths. We highly recommend training a diverse group of young adult facilitators to actively engage youth (Turuba et al., 2022). To help make youths feel valued, sessions should be prefaced by emphasizing the importance of their active role in research and hearing their lived experiences to create meaningful real-life applications.

## Conclusion

Online-based modalities can create challenges and opportunities to effectively engage youth in virtual settings. This is especially relevant in our post-pandemic world, as youth have migrated more towards communicating through online platforms and are often less comfortable communicating in-person. Integration of virtual communication strategies to research helps meet youth where they are in a comfortable setting to promote rich engagement. It also supports a more inclusive recruitment strategy to obtain diverse perspectives from harder to reach youth. To develop a substance use education plan that directly serves the needs of youths, we continuously engaged youth as knowledge experts while upholding autonomy, cultural humility, and prioritizing safety (Lansdown, 2010; Lundy, 2007; Ozer et al., 2018). We identified challenges and facilitators to achieving our goal of prioritizing representation of youths and real-life knowledge translation while embracing lived experiences to enhance youth engagement practices to best meet their needs.
